# Psychosocial Aspects and Diabetes Technology – Head to Head or Hand in Hand?

**DOI:** 10.17925/EE.2016.12.01.35

**Published:** 2016-03-15

**Authors:** Katharine D Barnard, Jill Weissberg-Benchell

**Affiliations:** 1 Faculty of Health & Social Sciences, Bournemouth University, Bournemouth, UK; 2 Northwestern University’s Feinberg School of Medicine, Ann and Robert H. Lurie Children’s Hospital of Chicago, Chicago, US

**Keywords:** Diabetes technology, psychosocial, quality of life, Advanced Therapeutics and Technologies in Diabetes (ATTD)

## Abstract

Diabetes technologies have progressed rapidly over recent years with a dedicated conference entering its 10th year, stronger and larger than ever. The long-awaited automated insulin delivery systems represent the latest devices in engineering excellence however it is important that we do not lose sight of the fact that there is a person at the end of this technology, simply wanting a better life with diabetes with reduced diabetes burden. This commentary explores the relationship between technology and the psychosocial aspects of that technology in the context of user experience, clinical guidelines and the inclusion of psychosocial aspects alongside medical outcomes in research trials.

The recent Advanced Therapeutics and Technologies in Diabetes (ATTD) conference held in Milan, Italy was a timely reminder of how the mechanics and engineering precision involved in diabetes technologies can sometimes seem to be on the polar opposite end of the spectrum from the psychosocial aspects of these technologies. A review of the latest devices, such as data sharing continuous glucose monitoring systems, sensor augmented insulin pump therapy, and artificial pancreas algorithms (if that’s what we’re calling it – other names such as automated insulin delivery or closed loop also apply) reminds us of our amazing technological progress. Psychology, on the other hand, moves at a slower pace, and is often known as the study of the glaringly obvious. Yet, without assessing the psychosocial aspects of a person’s lived experience with diabetes technologies, a gulf between the intended use and the actual uptake and continued use of these devices remains. This is where the ‘study of the glaringly obvious’ comes into its own and provides valuable insights into the facilitators and barriers of technology use. The ultimate goal of assessing the psychosocial aspects of diabetes technology is to optimise outcomes – both biomedical and psychosocial – for people living with diabetes and for those who love them.

It was refreshing to see the ATTD embrace this union, as there was a growing focus on the psychosocial aspects of diabetes technology, with researchers around the globe presenting data and intervention approaches aimed at improving psychosocial outcomes for people living with diabetes. This reflects the growing awareness that maintaining the status quo regarding technology uptake and continued use is simply not an option. Lessons must be learned from the introduction of previous technologies such as continuous glucose monitoring (CGM). There is little doubt that when used effectively and continuously the benefits of CGM in reducing glycaemic variability, improving HbA_1c_ and improving self-efficacy and quality of life are clear. Yet, the reality is that very few people who are eligible to use this technology actually do so.^1^ There are several contributory factors for this gap between improved outcomes and device uptake. While it is widely recognised that latest generation CGMs are far improved than older devices, the current uptake rates may be influenced by negative past experiences and mis-perceptions hung-over from earlier systems.

New technologies must be developed with the end user experience in mind, not simply in terms of human factors (how an individual engages with the device itself) but also in terms of how each user and their families are able to incorporate the technology into their everyday lives to minimise diabetes-related burden, improve outcomes and optimise quality of life. Consistent with this goal, Dr Aaron Kowalski suggested that psychosocial outcomes should become a key factor in assessing new technologies, such as artificial pancreas systems.^2^ In fact, he suggested that “Diabetes Happiness" must be a part of the assessment of technology outcomes. Specifically, he suggested that systems must offer patients a sense of night time security, prevent headaches that result from low blood sugars, decrease the burden of the daily regimen demands, improve sleep quality and quantity, and offer the possibility of “unimaginable joy" to all users.

In keeping with this new focus on psychosocial outcomes, the Best Practice Paediatric Tariff for Diabetes^3^ and more recently the National Institute for Health and Care Excellence (NICE) guidance for children and young people with diabetes^4^ both incorporate psychosocial aspects of diabetes self-management within their guidance, reflecting that this is a crucial component alongside device and biomedical aspects of care. The growing impetus on effective use of new technologies to support optimal diabetes self-management is further highlighted in the publication today of the new NICE guidelines on sensor augmented pump therapy (SAP) for individuals with Type 1 diabetes (T1D). The Diagnostic Assessment Report (DG21)^5^ supplements NICE guidance TA151^6^ on insulin pump therapy and possibly opens the door for greater use of SAP However, access remains limited since only people experiencing episodes of disabling hypoglycaemia despite optimal management with insulin pump therapy can qualify. Therein lies a fundamental question however. What does ‘optimal management’ look like and how is it measured? The guidance further states that users must agree to use the sensors for at least 70% of the time, understand how to use SAP (seems bizarre as surely one would like to believe that appropriate training would be given?) and agree to use the system whilst having a structured education programme on diet, lifestyle and counselling. Wait a minute. What does that actually mean?

With the availability of structured education abysmally poor^7^ and at the risk of being controversial, is this simply the equivalent of a ‘stealth tax’ to restrict access to SAP technology and force people with T1D to jump through ever increasing numbers of hoops to be able to access and benefit from it? Who will provide the education on diet, lifestyle and counselling? What will the content of that structured education be? To be even more controversial, how can individuals who are struggling with disabling hypoglycaemia use SAP without intensive support when, as an example, the University of Virginia’s artificial pancreas (AP) team (which provides intensive education, supervision, and monitoring) reported during this ATTD meeting that fully 30% of their study participants did not use the system consistently.^8^ These 14 individuals wore their AP system for 6 months with all of the support a funded research study could provide and yet 30% of their participants did not use the system consistently. Sadly, the reasons for that discontinued use were not reported. Gathering information about the reasons for discontinued use are vital for all technology trials and will be key to helping create programmes that actually increase the chance that patients will be successful in their use of technology.

As new automated insulin delivery systems are developed, there is a considerable effort to learn these lessons and research teams around the world, regulatory approvals bodies, industry, advocacy groups and academia are pulling together to ensure that psychosocial outcomes are effectively assessed alongside safety and biomedical outcomes. The views of healthcare professionals are being sought alongside those of people living with T1D to ensure that when devices are brought to market and available, there is a supportive clinical environment and process to support uptake and continued use.

It is recognised that the introduction of these new systems represents a ground-shift in what diabetes technology will do for the individual user. All devices to date support self-management, however automated insulin delivery systems will take much of the self-management tasks away from the individual. This brings both positive and potentially negative impacts psychosocially in terms of quality of life, control over diabetes, time required to manage diabetes and potential reduction in diabetes-related burden.

Back to the ATTD, in his overview of the importance of understanding the psychosocial impact of automated insulin delivery (AID) systems, Dr Richard Bergenstal asked attendees to consider whether or not the patient’s needs are at the centre of the development of AID systems.^9^ He further challenged the audience to work toward improving their collaborative relationship with their patients by being empathic listeners. He lauded the Food and Drug Administration (FDA) for creating a patient engagement advisory committee to help gather information regarding patient’s needs, experiences and perspectives. Questions regarding the patient’s experiences regarding technology’s comfort and tolerability were raised as was the user’s ability to trust the technology, whether using it offered a sense of hope and whether it impacted family relationships. During the same session, Dr. Katharine Barnard asked the audience to consider the balance between outcomes versus cost and suggested that both sides of the equation depend upon the stakeholder.

So that brings us back to perhaps the most important question of all. How do we best support individuals with T1D to use technologies to their best advantage, whilst minimising the burden on everyday living? Let’s hope we can present the answer to that question in relation to AID systems at ATTD conferences in the not too distant future. Watch this space.

## References

[1] Bode B. W., Beck R. W., DuBose S. N. (2012). T1D Exchange Clinic Network. A comparison of users and nonusers of real- time continuous glucose monitoring with type 1 diabetes in the type 1 diabetes exchange. J Diabetes Sci Technol.

[2] Kowalski A. (2016). It is Time to Move to Time in Range as Measure of Glycemic Control.

[3] (2012). Best Practice Tariff for Paediatric Diabetes. http://www.evidence.nhs.uk/document?ci=http%3a%2f%2fwww.diabetes.nhs.uk%2fnews_and_events%2ffirst_ever_mandatory_care_standards_bring_hope_of_improved_care_for_children_and_young_people_with_diabetes1%2f%3ffromsource%3dnelm&returnUrl=Search%3fq%3dbest%2bpractice%2btariffs&q=best+practice+tariffs.

[4] (2015). Diabetes (type 1 and type 2) in children and young people: diagnosis and management [NG18]. http://www.nice.org.uk/guidance/ng18.

[5] (2016). Integrated sensor-augmented pump theray for systems managing blood glucose levels in type 1 diabetes the MiniMed Paradigm Veo system and the Vibe and G4 Platinum CGM system) [DG21]. http://www.nice.org.uk/guidance/dg21.

[6] (2008). Continuous subcutaneous insulin infusion for the treatment of diabetes mellitus [TA151]. http://www.nice.org.uk/guidance/ta151.

[7] (2010). Emotional and Psychological Support and Care in Diabetes. http://www.diabetes.org.uk/About_us/What-we-say/Diagnosis-ongoing-management-monitoring/Emotional-and-Psychological-Support-and-Care-in-Diabetes/.

[8] University of Virginia’s artificial pancreas team (2016). Plenary Session: Closing the Loop. And Dr. Kovatchev’s talk was entitled: JDRF Multi-center 6-month trial of 24/7 closed loop control.

[9] Bergenstal R. (2016). Artificial Pancreas Psychosocial Measures Project.

